# Thymol Chemotype *Origanum vulgare* L. Essential Oil as a Potential Selective Bio-Based Herbicide on Monocot Plant Species

**DOI:** 10.3390/molecules25030595

**Published:** 2020-01-29

**Authors:** Daniela Gruľová, Lucia Caputo, Hazem S. Elshafie, Beáta Baranová, Laura De Martino, Vincent Sedlák, Zuzana Gogaľová, Janka Poráčová, Ippolito Camele, Vincenzo De Feo

**Affiliations:** 1 Department of Ecology, Faculty of Humanities and Natural Sciences, University of Prešov, 17. Novembra 1, 08001 Prešov, Slovakia; beata.baranova@unipo.sk; 2 Department of Pharmacy, University of Salerno, I-84084 Fisciano, Italy; lcaputo@unisa.it (L.C.); ldemartino@unisa.it (L.D.M.); defeo@unisa.it (V.D.F.); 3 School of Agricultural, Forestry, Food and Environmental Sciences, University of Basilicata, Viale dell’Ateneo Lucano 10, 85100 Potenza, Italy; hazem.elshafie@unibas.it (H.S.E.); ippolito.camele@unibas.it (I.C.); 4 Department of Biology, Faculty of Humanities and Natural Sciences, University of Prešov, 17. Novembra 1, 08001 Prešov, Slovakia; vincent.sedlak@unipo.sk (V.S.); zuzkagogalova@gmail.com (Z.G.); janka.poracova@unipo.sk (J.P.)

**Keywords:** monocots, dicots, thymol chemotype, phytotoxicity, antimicrobial activity, antifungal activity

## Abstract

Searching for new bio-based herbicides is crucial for decreasing chemical pollution, protecting the environment, and sustaining biodiversity. *Origanum vulgare* is considered a promising source of essential oil with herbicidal effect. The mode of action is not known. The present study focused on (1) comparison of phytotoxic activity of *Origanum vulgare* EO on monocot (*Triticum aestivum* and *Hordeum vulgare*) and dicot species (*Lepidium sativum* and *Sinapis alba*); (2) and evaluating other antimicrobial biological activities against phytopatogen bacteria (*Clavibacter michiganensis*, *Pseudomonas syringae* pv. *phaseolicola*, *Pseudomonas savastanoi,* and *Xanthomonas campestris*); antifungal activity against *Monilinia fructicola*, *Aspergillus niger*, *Penicillium expansum*, and *Botrytis cinerea*; cytotoxic activity and antioxidant activity. According to the GC/MS analyses, the EO belongs to the thymol chemotype *O. vulgare* with its high content of thymol (76%). Germination of all four species was not influenced by EO. The phytotoxic effect was statistically significant in the monocot species, while in the dicot species the opposite was observed—a stimulation effect, which was also statistically significant. Strong biological activity of *O. vulgare* EO was noted on all phytopatogen bacteria and fungi in the highest dose. Cytotoxic activity showed an IC_50_ = 50.5 μg/mL. Antioxidant activity showed an IC_50_ = 106.6 μg/mL after 45 min experimental time. Based on the presented results, it is possible to conclude that thymol chemotype *O. vulgare* essential oil could be potentially used as a herbicide with selective effects on monocot plant species.

## 1. Introduction

Herbicides are still dominant tools used in weed management. A herbicide with a new mode of action (MOA) has not been introduced as a commercial product for more than 25 years [1]. Thus, the discovery of new herbicides with new MOAs is particularly urgent [2]. Based on the ecological and political press on environmental protection, there is new attention on the search for natural compounds with herbicidal and/or pesticidal effect [3,4,5]. Plants and some animals have developed specific mechanisms to protect themselves against enemies by production of secondary metabolites, which could be the source of new herbicides and/or pesticides. Moreover, it is important to evaluate the safety of new herbicides and pesticides because many of them produce adverse neurological effects [6].

Essential oils (EOs) are complex mixtures of mostly terpenoids as plant secondary metabolites. It has been recognized that various EO components act as multi-target molecules exerting several MOAs in the recipient-organism [7,8], that can be beneficial compared to conventional herbicides which induce resistance [9]. Single EO molecules are able to cross the cell wall and directly interact with the plant plasma membrane, which is one of the potential cellular targets of EOs. It is known that while monoterpenes disturb the lipid organization and/or domain formation, phenylpropanoid could interact with membrane receptors [9].

The use of plant EOs as possible synthetic substitutes has attracted a high interest from many scientists all over the world [5,10,11,12]. Volatile terpenes and EOs have been reported as regulators of germination and growth of other species in several ecosystems [13].

*Origanum vulgare* L. (*Lamiaceae*) is an aromatic plant, commonly known as oregano, widespread in Mediterranean region as well as other European countries [14,15,16]. It is a rich source of EO which has proven to possess a wide variety of biological activities. Variable effects of oregano species, not only *O. vulgare* but also *O. heracleoticum* L., *O. majorana* L., *O. acutidens* (Hand.-Mazz.) Ietsw, and *O. onites* L. have been considered to be due to their bioactive ingredients such as thymol and carvacrol, which act in a synergistic manner, as well as others such as linalool, γ-terpinene, or cis- and trans-sabinene hydrate [17,18,19,20,21]. The composition of EOs from the same plant species can vary considerably, depending on the several factors such as growth conditions of the plant and the genetics of the variety of the plant species [2]. *O. vulgare* EO showed promising antibacterial, antifungal, and antiviral activities against several phytopathogens [11,22,23,24,25]. Oregano EO was able to inhibit significantly some phytopathogens such as *Botrytis cinerea* Pers., *Penicillium expansum* Link, *Phytophthora citrophthora* (R.E. Sm. and E.H. Sm.) Leonian, *Rhizopus stolonifera* Vuillemin, *Aspergillus niger* van Tieghem, *Fusarium oxysporum* von Schlechtendal, *Sclerotinia sclerotiorum* (Lib.) de Bary, *Staphylococus aureus* Rosenbach, *Clavibacter michiganensis* corrig. (Smith), and *Xanthomonas vesicatoria* (Doidge) Dowson [24,25,26].

*O. vulgare* as well as relative species as *O. heracleoticum* L., *O. majorana* L., and *O. acutidens* have been tested for its phytotoxic effect on dicotyledonous model plant species (*Lepidium sativum* L., *Raphanus sativus* L., *Lactuca sativa* L., and *Solanum lycopersicum* (L.) H. Karst.; *Amaranthus retroflexus* L., *Chenopodium album* L., and *Rumex crispus* L.) [5,27]. Scientific studies noted potential phytotoxic effect of *O. vulgare* which were tested on various dicot plant species (*Sinapis arvensis* L., *Phalaris canariensis* L., *Lepidium sativum* and *Raphanus sativus*.; *Abutilon theophrasti* Medik., *Agrostemma githago* L., and *Medicago sativa* L.) [13,25,28,29].

However, monocot plant species, for example cereal grains, present a staple food that provides more food energy worldwide than any other type of crop. Corn (maize), wheat, and rice—in all of their varieties—account for 87% of all grain production worldwide [30]. Crop protection of such important food sources then depends on selective modes of action in the use of used herbicides. On the other hand, there are a lot of weed plant species, which are monocot (*Poa annua* L., *Hordeum jubatum* L., *Eleusine indica* L., *Bromus tectorum* L. etc.) and have negative impact on dicot crops. Producing herbicidal EOs with optimal constituents and MOA would be attractive, but little is known of the contributions and interactions of the different components of the EOs that are sold as herbicides [2]. The regulation of both existing and new herbicides has required more extensive toxicology and environmental testing [31].

The EO of one species can vary, depending on chemotype. A chemotype is a “chemical race”, meaning that plants sharing the same botanical name (same genus and species) can exhibit a completely different chemical composition [32].

Based on our knowledge, there is no evidence in the scientific literature that *O. vulgare*—thymol chemotype EO was tested for potential phytotoxic effects with a selective effect on monocot and dicot model plant species. All previous studies focused on dicot species.

The main aim of the current study was to focus on the comparison of potential selective herbicidal activity of oregano EO. Additional bio-chemical and biological assay as GC/MS analysis of EO, antimicrobial and antifungal activity against some phytopathogens, as well as cytotoxic and free radical-scavenging capacity of oregano EO have been provided, to study the complexity of its biological activity. If there is an assumption that the EO of oregano could be used in agrosystems as a bio-based herbicide, it is very important to mention that agrosystems present a complex ecological system, where all living beings are in interaction. It was very important to provide basic antimicrobial and antifungal activity on strains which occur naturally in agrosystems as well as to study their possible cytotoxicity on SH-SY5Y cells, considered as a model of neuronal cells, to evaluate their possible effects on human health. Finally, it was also important to test generally their antioxidant and cytotoxic activity to widen the knowledge about the influence of the EO–thymol chemotype.

## 2. Results

### 2.1. GC-MS Analyzes

Analyzes of commercially-available EO of *O. vulgare* L. allowed the identification of 42 components which presented 96.4% of total identified components (Table 1). The highest quantity reached thymol (76.0 %). Among the main compounds, we also identified p-cymene (5.7%), carvacrol (3.2%), linalool (2.6%), and γ-terpinene (2.5%). Based on the dominance of thymol, the tested oregano EO is thymol chemotype.

### 2.2. Phytotoxic Activity

#### 2.2.1. Germination

The influence of different doses of oregano EO on the seed germination of two dicot species (*Sinapis alba* and *Lepidium sativum*) and two monocot species (*Hordeum vulgare* and *Triticum aestivum*) was investigated (Table 2). The total number of evaluated seeds for each species was 30, made as a triplication of ten seeds in one petri dish. In general, the number of germinated seeds decreased with the increase of the applied EO dose. The highest number of germinated seeds of *S. alba* was 27 seeds after application, the two lowest EO doses (0.125 and 0.0625 µg/mL) was 22 seeds after the application of the highest dose EO (100 µg/mL). The lowest number of seeds was 21, evaluated after application of EO dose 2.5 µg/mL. In the control sample were 23 germinated seeds. After application of seven doses of EO (0.0625; 0.125; 0.25; 1.25; 5.0; 10.0, and 50.0 µg/mL), the number of germinated seeds was higher in comparison to the control, and after application of three doses (0.625; 2.5; 25.0, and 100 µg/mL) the number of germinated seeds was lower in comparison to the control.

The highest number of germinated seeds of *L. sativum* was 30 seeds, after application of the seven lowest EO doses (0.0625–5.0 µg/mL), which was also a higher number compared to the control. The lowest number of seeds was 27, evaluated after application of EO doses 10, 20, and 50 µg/mL, and 28 seeds after application of EO dose 100 µg/mL. Twenty-nine seeds were germinated in the control sample.

The highest number of germinated seeds of *H. vulgare* was 22 seeds after application of EO of dose 25 µg/mL. The lowest number of germinated seeds was 6 after application of the lowest EO dose 0.0625 µg/mL. In the control sample, 17 seeds were germinated. In five doses of EO (25; 10; 2.5; 1.25, and 0.25 µg/mL), the number of germinated seeds was higher compared to the control, and in six doses (100, 50, 5, 0.625, 0.125, and 0.0625 µg/mL) the number of germinated seeds was lower compared to the control.

The highest number of germinated seeds of *T. aestivum* was 24 seeds after application of EO of dose 1.25 µg/mL. The lowest number of germinated seeds was 18 after application of EO with dose 0.625 and 0.25 µg/mL. In the control sample, 18 seeds were germinated. After application of all EO doses, the number of germinated seeds was the same or higher than in control sample.

Slight stimulation, as well as an inhibition effect, was noted in different doses. No significant effect of oregano EO-influenced germination of four species was confirmed.

#### 2.2.2. Root Length

The influence of different doses of oregano EO on root elongation of two dicot species (*S. alba* and *L. sativum*) and two monocot species (*H. vulgare* and *T. aestivum*) was investigated. Sensitivity with an inhibition effect by oregano EO was noted to be higher in monocot species in comparison to dicot species (Table 3). After application of ten doses of origanum EO on *Sinapis alba*, we noted longer root length (1.8–3.2 cm) than in the control sample, and in six of them (100–5 µg/mL and 0.0625 µg/mL) there were significant differences. The dose of 2.5 µg/mL was the only dose where roots were shorter (1.2 cm) in comparison with the control (1.5 cm). In this case, the influence of EO was not significant. A similar observation was noted after the application of different doses of oregano EO on seeds of *L. sativum*. From the lowest dose (0.0625 µg/mL) to the second highest (50 µg/mL), the roots were longer (7.2–9.0 cm) than in the control sample (4.9 cm). Except for one dose (10 µg/mL), the influence of EO was significant. The highest dose (100 µg/mL) influenced root length negatively (3.6 cm) with statistical significance. The opposite effect was noted in monocot species, where after the application of eight doses of EO (100–25 µg/mL and 1.25–0.0625 µg/mL) on *H. vulgare* seeds, an inhibition effect was noted, and in six doses was significant. In three doses root lengths were slightly higher (3.3–3.5 cm) compared to the control (3.2 cm), but without statistical significance. The effect of oregano EO on *T. aestivum* root length was very similar to the other monocot species *H. vulgare*. Except for one dose (5 µg/mL), the root length was lower (1.1–2.5 cm) compared to the control (2.6 cm), and in six doses there were significant differences.

Generally, a significant stimulation effect of oregano EO was noted in dicot species *L. sativum*. A significant inhibition effect of oregano EO was noted in three species (*L. sativum*, *H. vulgare*, and *T. aestivum*), at the highest dose. The same dose (100 µg/mL) was significantly stimulative on *S. alba*. A significant stimulation effect on root elongation in dicot species was noted in four tested doses (50 µg/mL, 25 µg/mL, 5 µg/mL, and 0.0625 µg/mL). A significant inhibition effect on monocot species was evaluated in five different doses (100 µg/mL, 25 µg/mL, 1.25 µg/mL, 0.125 µg/mL, and 0.0625 µg/mL) (Table 3).

### 2.3. Cytotoxic Activity

MTT assay is the most applied method for screening new drugs, either from natural or synthesis origin [33] and it is based on the reduction of MTT due to NADPH-dependent cellular oxidoreductase enzymes. Live cells reduced MTT to formazan while the dead or inactive cells are not capable to do this [34]. IC_50_ defined as dose of EO required to reduce the cell viability by 50% [35].

**Figure 1:**
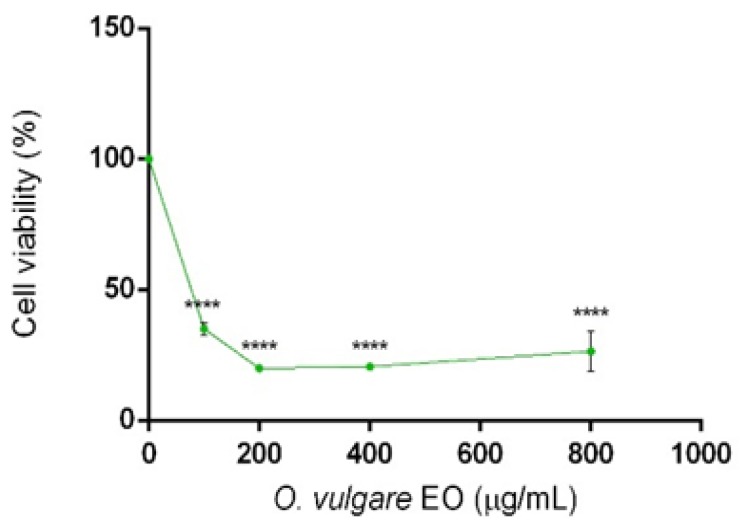
Percentage of cell viability after 3-(4,5-dimethylthiazol-2-yl)-2,5-diphenyl tetrazolium bromide (MTT) assay. Cells were treated with different doses (800–100 µg/mL) of *Origanum vulgare* EO for 24 h and solvent (DMSO, 0.1%) alone. Data are the mean ± SD of three experiments **** - *p* < 0.0001.

*O. vulgare* EO was evaluated for its possible cytotoxic activity on human neuroblastoma cell line (SH-SY5Y) and showed an IC_50_ = 50.5 μg/mL (Figure 1).

### 2.4. In Vitro Antibacterial Effect

**Figure 2:**
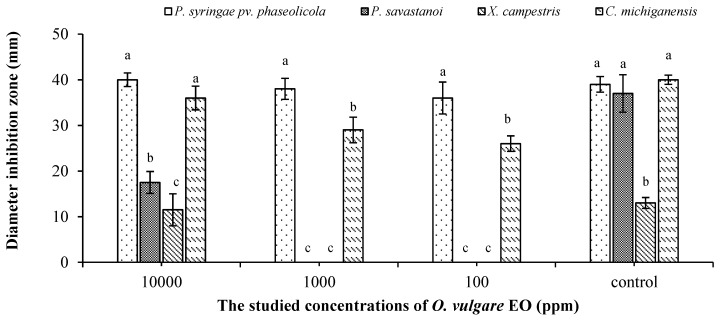
Antibacterial activity of *Origanum vulgare* EO Bars with different letters for each tested EO dose indicate mean values significantly different at *p* < 0.05 according to Tukey (B) test. Data are expressed as mean of three replicates ± SD.

The studied oregano EO showed a promising antibacterial effect on four tested phytopathogenic bacteria in a dose-dependent manner compared to the control (Figure 2). In particular, *P. syringae* pv. *phaseolicola* was inhibited completely by all tested doses of oregano EO. *P. savastanoi* and *X. campestris* were inhibited only with the highest tested dose (10,000 ppm).

### 2.5. In Vitro Antifungal Effect

**Figure 3:**
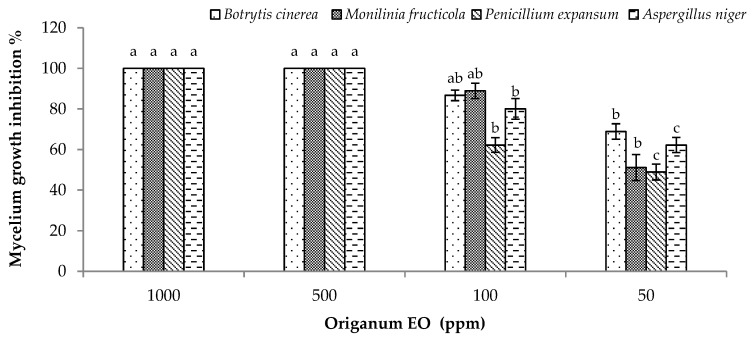
Antifungal activity of *Origanum vulgare* EO. Bars with different letters for each tested fungus indicate mean values significantly different at *p < 0.05* according to Tukey B test. Data are expressed as mean of three replicates ± SD.

Results showed that all tested doses of oregano EO were able to inhibit the growth of phytopathogenic fungi (*Monilinia fructicola* (G.Winter) Honey, *A. niger*, *P. expansum,* and *B. cinerea*) (Figure 3). In particular, the highest inhibition activity was observed in the case of 1000 and 500 ppm against the 4 tested fungi. Whereas, the other 2 tested doses (100 and 50 ppm) showed moderate activity in a dose dependent manner (Figure 3).

### 2.6. Free Radical-Scavenging Capacity

Anti-radical scavenging activity was tested by the 2.2-diphenyl-1-picrylhydrazil (DPPH) model system and expressed as absolute percentage of DPPH remaining in solution. In its radical form, DPPH has an absorption band at 515 nm, which disappears upon reduction by an antiradical compound. The absorbance of DPPH without the antioxidant (control sample) was used as the baseline measurement. The scavenging activity was expressed as the half maximal effective dose (EC_50_) of the sample, in terms of µg/mL, necessary to inhibit the DPPH radical activity by 50%.

In Table 4, several doses of *O. vulgare* EO were reported, at three incubation times.

**Figure 4:**
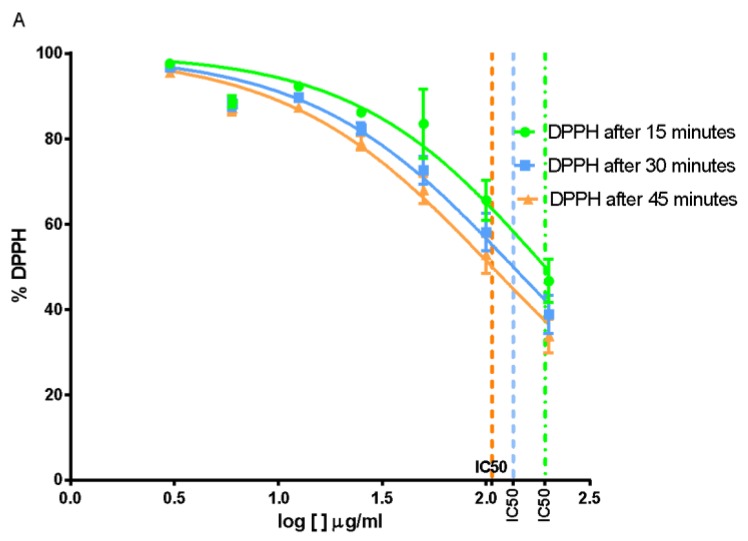
Percentage of DPPH in solution after 15, 30, and 45 min of treatment with different doses of *Origanum vulgare* EO.

From statistical analysis, *O. vulgare* EO showed an IC_50_ = 192.3 μg/mL after 15 min, IC_50_ = 134.9 μg/mL after 30 min and IC_50_ = 106.6 μg/mL after 45 min (Figure 4).

## 3. Discussion

In the studies where oregano EO was tested on its phytotoxic activity, we have noted different species of oregano as well as different chemotypes. The relative species analyzed were *O. heracleoticum* with its high content of carvacrol (77.8%), *O. majorana* with the chemical composition of dominant components terpinen-4-ol 29.6%, δ-2-carene 20%, and camphene 13% [5], *O. onites* and *O. acutidens* with their high content of carvacrol (66% and 87%) [27,36]. High content of carvacrol (84%) was also evaluated in oregano hybrid: *O. vulgare* L. ssp. *virilidum* x *O. vulgare* L. ssp. *hirtum* (Link) Iestwart [37], which was tested on phototoxic activity. Two scientific works have published the results of phytotoxic activity of *O. vulgare* ssp. *hirtum* L. where the dominant compounds were identified in the first case as o-cymene 19.8%, thymol 27%, and carvacrol 19% [8], and in a second study as 1,8 cineol 36% and camphor 17% [36]. Compared to other studies which focused on the same species as our experiment (*O. vulgare*), the dominant compounds were identified as (1) (carvacrol 44% and p-cymene 42%) [38]; (2) carvacrol (34.0%), γ-terpinene (21.6%), and p-cymene (9.4%) [29], and unspecified species of *Origanum* (3) carvacrol (60%), p-cymene (15.5), and γ- terpinene (5%) [39]. In only one study the EO of *O. vulgare* had thymol (50.41%) as dominant compound, following by carvacrol (12.96%), 2-bornene (11.28%), z-terpinen (8.80%), and o-cymene (6.68%) [29]. In the commercial sample of *O. vulgare* was identified dominant compound thymol with the amount of 76%. The chemical composition of the present sample differs from others EO in previous similar studies.

The design of published studies is slightly different but basically used similar principles. All studies focused of testing the phytotoxic activities on dicot model plant species and the results showed strong activity in each of these studies, although different EO doses were used. The herbicidal effect was evaluated on seed germination as well as on root length respective to seedling development [5,8,27,28,36,38].

The only previous study evaluated the potential herbicidal effects of *O. onites* and *O. vulgare* ssp. *hirtum* EO against monocot plant species (*Echinochloa crus-galli* and *Oryza sativa*) compared to the other tested plant species; both monocot species were less sensitive [36]. The molecular structure of each component can be responsible for the specific MOA as was previously mentioned [9]. Developing a selective herbicide, even if some data are promising, is difficult in the moment. It is well known that the major problem with EO is its volatility. The final formulation of the product is therefore of almost importance. One solution of the volatility problem could be the controlled release by using encapsulation. Encapsulation conserves and protects EOs from outside aggression, which is useful for applications in agronomy [32].

It is known that many herbicides, such as glyphosate, are associated with a cytotoxic and demyelinating effects [40]. In addition, several pesticides such as organophosphates, carbamates, and organochlorine have a direct effect on nervous system [6]. For this reason, our current research has also evaluated the possible cytotoxic effect of *O. vulgare* EO against Central Nervous System using SH-SY5Y cells.

We obtained promising results, for the first time, for *O. vulgare* EO from Slovakia origin against the SH-SY5Y cell line. Other *O. vulgare* EOs from different origins such as Jordan, Chile, and Italy showed less activity against other tumor cell lines such as adenocarcinoma (MCF7), human colon adenocarcinoma (HT-29), and human hepatocellular carcinoma (HepG2) cell lines, respectively [23,41,42]. In fact, after incubation for 72 h *O. vulgare* EO from Jordan showed IC_50_ = 30.1 μg/mL against MCF7 cells [34]. After 72 h, the percentage of HT-29 cell growth inhibition was 60.8% at a dose of 50 mg/mL of EO from Chile [35]; in this study, a treatment of 100 μg/mL in SH-SY5Y cells for 24 h showed an inhibition of 64.9%. The antiproliferative activity of *O. vulgare* EO was tested also in HepG2 cell with an IC_50_ of 236 μg/μL at an amount 50-times greater than our results [21]. However, our IC_50_ values and those reported in previous studies indicated that *O. vulgare* EO is not cytotoxic as judged by the criterion set by the National Cancer Institute, because its IC_50_ is <20 µg/mL [43].

The antibacterial activity of the EOs is dependent on the composition and dose of the EO, the type and dose of the target microorganism [44]. Oregano EO showed promising antibacterial, antifungal, and antiviral activities against several phytopathogens [11,25]. The antimicrobial activity can be explained by the chemical composition of phenolic compound present in oregano [24]. In particular, carvacrol and thymol are phenolic compounds with similar structures previously showed antifungal activity against many pathogenic fungi [5,25]. These phenolic compounds have a lipophilic character, acting in the cell wall and interfering with membrane-catalyzed enzymes and with enzymes responsible for energy and protein production, causing cell death [11,24]. Several studies have focused on the bioactive constituents of oregano plant as well as its EO [22,23,24].

When the cell endogenous detoxifying systems are no longer able to scavenge reactive oxygen species (ROS), oxidative stress arises. This stressed condition can damage lipids, proteins, and nucleic acids, leading to irreversible damages and cell death: For this motive, the quantity of ROS inside the cell increases endogenous and exogenous ROS production [45]. The antioxidant properties of *O. vulgare* EO have been extensively investigated using 2.2-diphenyl-1-picrylhydrazil (DPPH) method. A slight antiradical power of oregano EOs with percentage values from 2.85% to 3.68%, increasing with doses (7–9 mg/mL), was reported [46]. Other researchers have documented a similar low activity with EC_50_ value of 1509.1 ± 119.0 µg/mL corresponding to 0.05 antioxidant activity index (AAI) [47]. Higher inhibition activity (93%) of the synthetic free radical at the highest tested EO dose (50 µL/mL) was obtained by Kačániová et al. [48]. Considerable scavenging activity (59.09% and 54.8%) at both tested EO doses (1000 and 100 ppm) was observed [49]. The quite good radical scavenging activity in comparison with data reported in literature could be due to a high percentage of thymol found in this oregano sample: It was reported that the antioxidant activity of thymol alone is 104.44 μg/mL [50].

## 4. Materials and Methods

### 4.1. Essential Oil

The studied EO was obtained from the company Calendula a.s. Slovakia, which is specialized for production variety of EOs for commercial use.

### 4.2. Tested Plant Seeds

Evaluation of potential phytotoxic effect of EO was evaluated on seeds of four species. We chose two dicotyledonous species as *Sinapis alba* L. (white mustard) and *Lepidium sativum* L. (garden cress) and two monocotyledonous species *Triticum aestivum* L. (common wheat) and *Hordeum vulgare* L. (barley). *S. alba* and *L. sativum* L. (cv. ’Dánska’) seeds were purchased from Zel Seed (Slovakia). Common wheat and barley were obtained from the Research Center in Malý Šariš (Slovakia).

### 4.3. Phytotoxic Assay

Phytotoxic assay followed the previously used method [25] with slight modifications. Two factors were taken into account in the experimental treatment: (i) Four test plants: (*S. alba* L., *L. sativum* L., *T. aestivum* L., and *H. vulgare* L.) and (ii) eleven different *O. vulgare* EO doses: (100–0.062 μg/mL). The tested EOs were dissolved in distilled water/acetone 99.5:0.5 and diluted to prepare the desired doses. Distilled water/acetone 99.5:0.5 was used as a control. Test seeds were surface sterilized in 95% EtOH for 15 s and rinsed triplicate in distilled water. Ten sterilized seeds were sown into each Petri dish (90 mm diameter) containing 5 layers of Whatman filter paper. In each Petri dish 7 mL of EO solutions of different doses or distilled water/acetone 99.5:0.5 was added. Each treatment was triplicated. The Petri dishes were kept in a growth chamber (20 ± 1 °C, natural photoperiod, Sanyo, MLR-351H). Evaluation of germination and the radicle length (cm) was measured after 120 h.

### 4.4. GC/MS Analysis

Samples of EOs were analyzed by a gas chromatography/mass spectrometry (GC/MS) at University of Prešov (Prešov, Slovakia). The analyses were done by Varian 450-GC gas chromatograph with 220-MS IT Mass Spectrometer (Varian, Inc., CA, USA). Separation of individual components was provided using a capillary column BR 5 ms (30 m × 0.25 mm ID, 0.25 μm film thickness; Bruker Daltonics Inc., MA, USA). Injector type 1177 was heated to a temperature of 220 °C. Injection mode was split less (1 μL of a 1:1000 n-hexane solution). Helium was used as a carrier gas at a constant column flow rate of 1.2 mL min^−1^. Column temperature was programmed in four steps: (1) 50 °C for 10 min, (2) 100 °C at 3 °C min^−1^, (3) isothermal for 5 min, and (4) 150 °C at 10 °C min^− 1^. The total time for analysis was 87.67 min. The MS trap was heated to 200 °C, manifold 50 °C and transfer line 270 °C. Mass spectra were scanned every 1 s in the range 40–650 *m*/*z*. The retention indices were determined in relation to the Rt values of a homologous series of n-alkanes (C10–C35) under the same operation conditions. Constituents were identified by comparison of their retention indices (RI) with published data in different literature. Further identification was made by comparison of the mass spectra with either those stored in NIST 02 library or with those from the literature [51,52]. Relative doses of components were evaluated as percentage of peak area normalization.

### 4.5. Antibacterial Activity

#### 4.5.1. Tested Bacteria

The tested bacteria were *C. michiganensis*, *P. syringae* pv. *phaseolicola*, *P. savastanoi,* and *X. campestris*. All bacteria were conserved in the collection of the School of Agricultural, Forestry, Food, and Environmental Sciences (SAFE), Basilicata University, Potenza, Italy.

#### 4.5.2. Bactericidal Assay

The antibacterial test of the studied EO was investigated following the disc diffusion method [12,53]. The above tested bacterial strains have been cultured on King B nutrient media (KB) [54]. The bacterial suspensions were prepared in sterile distilled water at 106 CFU/mL doses. Mixtures of bacterial suspensions in soft agar (0.7%) were prepared at 9:1; (*v*/*v*) and then 4 mL of this mixture was poured into each Petri dish (90 mm). Tween 20 (0.2%) was added to each EO mixture to avoid the bacterial cellular aggregation at the following doses: 10000, 1000, 100, and 10 ppm. Four discs (6 mm-OXOID) were placed on the surface of Petri dishes and 15 µL from different doses of the studied EO was applied per each disc and the bactericidal effect was evaluated by measuring the diameter of inhibition zone (mm) in comparison to tetracyclin antibiotic (1600 µg.ml^−1^). The growth inhibition percentage (GIP) was calculated using the following Equation (1):

(1) $$GIP=100-\frac{\left(GC-GT\right)}{GC}\times 100$$

where: GIP represents the bacterial inhibition percentage, GC the average diameter of bacterial grown in plate (control) in mm and GT the average diameter of inhibition zone in mm. The test was repeated twice with three replicates.

### 4.6. Antifungal Activity

#### 4.6.1. Tested Fungi

Four serious post-harvest phytopathogenic fungi (*B. cinerea*, *M. fructicola*, *A. niger*, and *P. expansum*) were tested for the antifungal activity assay.

#### 4.6.2. Fungicidal Assay

The fungicidal activity of the tested oregano EO was evaluated following the methods [24,55] at three different doses 1000, 500, 100, and 50 ppm incorporated directly into Potato Dextrose Agar (PDA) medium at 45 °C. A fresh fungal disk (Ø 0.5 cm) was inoculated in the center of Petri dish. All plates were incubated at 22 ± 2 °C for 96 h under darkness and the diameter of fungal mycelium growth was measured in mm. PDA plates without any treatment were inoculated only with fungal disks as negative control (–ve). Fungi toxic effect was expressed as percentage of mycelium growth inhibition (PGI %) compared to (–ve) control using the formula [24,56] (Equation (2)):

(2) $$PGI\left(\%\right)=\frac{100\times \left(GC-GT\right)}{GC}$$

where PGI is the percentage of growth inhibition, GC is the average diameter of fungal mycelium in PDA (Control), and GT is the average diameter of fungal mycelium on the oil-treated PDA dish.

### 4.7. Free Radical-Scavenging Capacity

The antiradical activity of the EO was determined using the sTable 1,1-diphenyl-2-picrylhydrazyl radical (DPPH), according to the method [57] with some modifications [58]. In its radical form, DPPH has an absorption band at 517 nm, which disappears upon reduction by an antiradical compound. Briefly, an aliquot of the MeOH solution containing different amounts of the EO was added to a DPPH solution (7.6 × 10^−5^ M), prepared daily, kept in the dark when not used, to have a final volume of 1 mL in a straight-sided cuvette, optically clear container for holding liquid samples in a spectrophotometer. An equal volume of the DPPH alone was added to control tubes. Absorbance at 515 nm was measured on Multiskan Spectrum Microplate Spectrophotometer (Thermo Fischer Scientific, Vantaa, Finland) after 15, 30, and 45 min; the doses of the extracts were 3, 6, 12.5, 25, 50, 100, and 200 μg/mL. For preparation of the standard curve, different doses of DPPH methanol solutions (10–60 μg/mL) were used. The DPPH dose (μg/mL) in the reaction medium was calculated from the following calibration curve, determined by linear regression (r2: 0.9993):

Absorbance (λ 515) = 0.0008 + 0.0118 × [DPPH](3)

The scavenging capability of test extracts was calculated using the following equation:

(4) $$DDPH\;scavenging\;activity\;\left(\%\right)=\frac{100\times \left[A\left(\lambda 515\right)C-A\left(\lambda 515\right)S\right]}{A\left(\lambda 515\right)C}$$

where A(λ515)C is absorbance of a control with no radical scavenger and A(λ515)S is absorbance of the remaining DPPH in the presence of scavenger. The IC50 value was defined as the dose of sample which reduced the initial DPPH.

#### 4.7.1. Ascorbic Acid

Ascorbic acid (Fluka Buchs, Switzerland) was dissolved in methanol. The solution (5 μg/mL) was used for a calibration curve of DPPH reduction and as a chemical reference in comparison to the antioxidant capacity of the extracts.

#### 4.7.2. Statistical Analysis

All experiments were carried out in triplicate. Data of each experiment were statistically analyzed using GraphPad Prism 6.0 software followed by comparison of means (two-way ANOVA) using Tukey’s multiple comparisons test, at the significance level of *p* < 0.05.

### 4.8. Cytotoxic Activity

#### 4.8.1. Cell Cultures

Human neuroblastoma (SH-SY5Y) cancer cells were cultured in Roswell Park Memorial Institute Medium (RPMI) supplemented with 1% L-glutamine, 10% heat-inactivated fetal bovine serum (FBS), 1% penicillin/streptomycin (all from Sigma Aldrich) at 37 °C in an atmosphere of 95% O_2_ and 5% CO_2_.

#### 4.8.2. MTT Assay

Human neuroblastoma cancer cells (SH-SY5Y) were plated (5 × 10^3^) in 96-well culture plates in 150 µl of culture medium and incubated at 37 °C in humidified 5% CO_2_. The day after, a 150 µl aliquot of serial dilutions of EOs (800–50 µg/mL) selected on the basis of our previous studies [59,60], their main component or extracts and their fractions were added to the cells and incubated for 24 h. DMSO alone was used as control. Cell viability was assessed through MTT (3-(4,5-dimethylthiazol-2-yl)-2,5-diphenyl tetrazolium bromide) assay. Briefly, 30 µl of MTT (5 mg/mL) was added and the cells incubated for additional 3 h. Thereafter, cells were lysed and the dark blue crystals solubilized with 30 µl of a solution containing 50%, *v*/*v*, *N*,*N*-dimethylformamide, 20%, *w*/*v*, SDS with an adjusted pH of 4.5. The optical density (OD) of each well was measured with a microplate spectrophotometer (Thermo Scientific Multiskan GO, Monza, Italy) equipped with a 520 nm filter. Cell viability in response to treatment was calculated as a percentage of control cells treated with DMSO at the final dose of 0.1% viable cells = (100 OD treated cells)/OD control cells modifications [61].

### 4.9. Data Analysis

#### 4.9.1. Germination Activity

Germination activity was expressed in total number as well as in average % of germinated seeds after exposure to particular doses of oregano EO. Student T-test counted in EXCEL was used to test the differences among experiments and control in the % of germinated seeds. Descriptive Statistic in PAST 2.17c [62] was used to depict distinctions in the germination activity in % among experiments and control.

#### 4.9.2. Roots Length

Minimal, maximal, mean values (+ standard deviation), and median of the root lengths of the control and experimental samples by particular doses of oregano EO were calculated using Univariate statistics in PAST 2.17c [62]. Descriptive Statistic in PAST 2.17c [62] was used to depict distinctions in the root lengths in cm among experiments and control. Student T-test counted in EXCEL was used to test the differences among experiments and control in the roots’ length, as well as to test mutual distinctions in the roots’ length among experiments with one another. Student T-test counted in EXCEL was used to test the differences in the number of roots among *Hordeum vulgare* and control, *Triticum aestivum* and control as well as *Hordeum vulgare* and *Triticum aestivum* each other.

## 5. Conclusions

Germination of all four species was not influenced by EO. Phytotoxic effect was statistically significant in monocot species, while in dicot species the opposite was observed—the stimulation effect, which was also statistically significant. Based on the results we can conclude that EO of *O. vulgare* presented diverse effects on monocot and dicot plant species in observation of its biological activity.

## Figures and Tables

**Table 1:** Identified components in *Origanum vulgare* EO.

No.	Name of Compound	KI^exp^	KI ^lit^	%	Identif.	
1.	α-pinene	938	936	0.2	KI, MS, S	M
2.	camphene	951	950	0.2	KI, MS	M
3.	sabinene	976	973	0.1	KI, MS	M
4.	β-pinene	980	978	0.3	KI, MS, S	M
5.	α-terpinene	1016	1013	0.7	KI, MS, S	M
6.	p-cymene	1020	1015	5.7	KI, MS, S	M
7.	1,8-cineole	1033	1024	0.6	KI, MS, S	MO
8.	(Z)-β-ocimene	1035	1029	T	KI, MS, S	M
9.	γ-terpinene	1060	1051	2.5	KI, MS, S	M
10.	terpinolene	1088	1082	T	KI, MS, S	M
11.	linalool	1098	1086	2.6	KI, MS, S	MO
12.	camphor	1121	1123	0.7	KI, MS, S	MO
13.	L-trans-pinocarveol	1130	1125	T	KI, MS	MO
14.	borneol	1152	1150	0.8	KI, MS, S	MO
15.	terpinen-4-ol	1160	1164	0.7	KI, MS	MO
16.	α-terpineol	1178	1176	0.4	KI, MS	MO
17.	carvone	1217	1214	T	KI, MS, S	MO
18.	carvotanacetone	1230	1220	T	KI, MS	MO
19.	thymol	1270	1267	76.0	KI, MS, S	MO
20.	carvacrol	1282	1278	3.2	KI, MS, S	MO
21.	eugenol	1333	1331	0.1	KI, MS	MO
22.	α-cubebene	1354	1355	0.3	KI, MS	S
23.	α-gurjunene	1411	1413	T	KI, MS	S
24.	α-himachalene	1449	1450	0.1	KI, MS	S
25.	humulene	1454	1455	T	KI, MS	S
26.	allo-aromadendrene	1461	1462	T	KI, MS	S
27.	β-guaiene	1490	1488	T	KI, MS	S
28.	valencene	1494	1494	0.1	KI, MS	S
29.	α-muurolene	1495	1496	T	KI, MS	S
30.	γ-cadinene	1512	1507	0.1	KI, MS	S
31.	calamenene	1513	1517	T	KI, MS	S
32.	β-cadinene	1520	1526	0.4	KI, MS	S
33.	α-calacorene	1534	1527	0.1	KI, MS	S
34.	elemol	1539	1541	T	KI, MS	SO
35.	caryophyllene oxide	1580	1578	0.4	KI, MS	SO
36.	globulol	1583	1589	T	KI, MS	SO
37.	cedrol	1598	1603	T	KI, MS	SO
38.	γ-eudesmol	1620	1618	T	KI, MS	SO
39.	allo-aromadendrene epoxide	1621	1623	0.1	KI, MS	SO
40.	tau.cadinol	1634	1633	T	KI, MS	SO
41.	tau.muurolol	1635	1633	T	KI, MS	SO
42.	cubenol	1636	1630	T	KI, MS	SO
	Total Identified			96.4		

T—traces less than 0.1%; KI^exp^—expected Kovats index predicted by software, KI^lit^—comparison of Kovats index with literature (Adams; Hochmuth); Identification:, MS—comparison of mass spectra with the literature, S–comparison with external standard, M—monoterpenes, MO—oxygenated monoterpenes, S-sesquiterpenes, SO—oxygenated sesquiterpenes.

**Table 2:** Total number and % of germinated seed of model organisms in comparison to control after exposition to particular doses of *Origanum vulgare* EO.

	Applied Doses of EO (µg/mL)											
	Control	100	50	25	10	5	2.5	1.25	0.625	0.25	0.125	0.0625
***Sinapis alba***												
Numb. GS	**23**	22	23	22	25	24	21	24	22	23	27	27
% GS	**76.67**	73.33	76.67	73.33	83.33	80.00	70.00	80.00	73.33	76.67	90.00	90.00
***Lepidium sativum***												
Numb. GS	**29**	28	27	27	27	30	29	29	30	30	30	30
% GS	**96.67**	93.33	90.00	90.00	90.00	100	96.67	96.67	100	100	100	100
***Hordeum vulgare***												
Numb. GS	**17**	9	13	22	17	16	19	17	16	19	13	6
% GS	**56.67**	30.00	43.33	73.33	56.67	53.33	63.33	56.67	53.33	63.33	43.33	53.33
***Triticum aestivum***												
Numb. GS	**18**	19	23	23	22	22	22	24	18	18	23	21
% GS	**60**	63.33	76.67	76.67	73.33	73.33	73.33	80.00	60.00	60.00	76.67	70.00

Numb. GS—number of germinated seeds; % GS—percentage of germinated seeds; table squares highlighted by lighter grey indicate lower number % of germinated seeds, highlighted by darker grey indicate higher number % of germinated seeds in comparison to control. Total number of treated seeds was 30. Each treatment was triplicated.

**Table 3:** Average roots length in cm of particular model organisms in comparison to control after exposition to different doses of *Origanum vulgare* EO.

		Applied EO Doses [µg/mL]											
		Cont.	100	50	25	10	5	2.5	1.25	0.625	0.25	0.125	0.0625
*Sinapis alba*	Min	**0.3**	0.6	0.5	0.3	0.5	0.6	0.3	0.1	0.1	0.1	0.1	0.1
	Max	**3.9**	7.2	6.5	10.1	6.1	10.6	5.7	5.4	4.6	4.3	5.5	5.5
	Mean	**1.5**	3.2	3.1	3.4	3.2	2.8	2.3	1.9	1.8	1.2	1.8	2.3
	SD	**0.9**	1.9	1.9	2.8	1.6	2.3	1.8	1.6	1.5	1.1	1.6	1.6
	Median	**1.3**	3.3	2.8	2.5	3.1	2.1	1.6	1.9	1.2	0.7	1.2	2.5
	Signif.		***	***	**	***	**						*
*Lepidium sativum*	Min	**0.3**	0.5	2.1	1.8	0.5	0.6	0.5	0.2	0.3	0.4	0.5	1.5
	Max	**9.6**	5.8	11.2	13.2	13.1	12.0	13.1	12.8	12.5	13.0	11.0	12.3
	Mean	**4.9**	3.6	7.5	9.0	5.7	8.4	7.2	8.2	8.3	7.9	7.1	7.6
	SD	**2.4**	1.3	2.3	3.3	3.6	3.7	3.5	3.2	3.5	3.7	2.6	2.7
	Median	**5.5**	4.0	8.1	10.3	5.3	10.2	8.3	8.6	9.0	9.0	7.8	8.1
	Signif.		*	***	***		***	**	***	***	***	***	***
*Hordeum vulgare*	Min	**1.6**	0.6	0.7	0.1	0.4	0.4	0.6	0.3	0.3	0.4	0.3	0.5
	Max	**4.7**	3.1	6.3	4.6	6.3	5.3	5.7	3.5	4.0	6.8	4.3	3.4
	Mean	**3.2**	1.9	3.2	1.8	3.3	3.4	3.5	1.9	1.9	2.8	2.4	2.1
	SD	**0.9**	0.8	1.8	1.5	1.8	1.3	1.7	1.0	1.1	1.7	1.3	0.8
	Median	**3.3**	2.1	3.5	1.7	3.4	3.5	3.7	2.0	1.7	2.7	2.3	2.1
	Signif.		**		**				***	***		*	***
*Triticum aestivum*	Min	**0.4**	0.2	0.5	0.3	0.2	0.9	1.1	0.3	0.3	1.5	0.3	0.5
	Max	**3.9**	1.8	3.2	2.7	3.8	4.8	4.0	2.40	3.2	3.5	2.5	3.2
	Mean	**2.6**	1.1	1.9	1.7	1.9	2.8	2.5	1.7	1.9	2.4	1.5	1.8
	SD	**0.9**	0.5	0.8	0.6	1.1	0.8	0.9	0.5	0.9	0.6	0.6	0.7
	Median	**2.8**	1.2	1.8	1.8	1.9	2.9	2.8	1.9	2.0	2.3	1.5	1.8
	Signif.		***	*	**				***			***	**

Table square highlighted by lighter grey indicates shorter average roots length in comparison to control, highlighted by darker grey indicates higher average roots length in comparison to control. * - *p* < 0.05, ** - *p* < 0.01, *** - *p* < 0.001. Each treatment was triplicated.

**Table 4:** Antioxidant activity of different doses of *Origanum vulgare* EO. Data are reported as mean ± SD of three replicates. DPPH: 2.2-diphenyl-1-picrylhydrazil.

Sample/Extract	(µg/mL)	Log (µg/mL)	DPPH (%) after 15 min	DPPH (%) after 30 min	DPPH (%) after 45 min
CTRL DPPH			100 ± 2.00	100 ± 2.84	100.00 ± 3.10
Oregano	3	0.477	97.61 ± 0.40	96.75 ± 0.74	95.47 ± 0.92
Oregano	6	0.778	88.70 ± 1.34	88.20 ± 1.57	87.20 ± 1.69**
Oregano	12.5	1.097	92.31 ± 0.54	89.67 ± 0.53	87.41 ± 0.79***
Oregano	25	1.398	86.22 ± 0.86 *	82.42 ± 1.35**	79.06 ± 1.66****
Oregano	50	1.699	83.52 ± 8.14****	72.64 ± 3.23****	68.00 ± 3.15****
Oregano	100	2.000	69.59 ± 4.72****	58.14 ± 4.46****	52.84 ± 4.30****
Oregano	200	2.301	46.66 ± 5.07****	38.89 ± 4.45****	33.80 ± 3.96****

* - *p* < 0.05, ** - *p* < 0.01, *** - *p* < 0.001, **** - *p* < 0.0001. Values followed by different stars are significant according to Tukey’s multiple comparisons test.
